# Northeast India Helminth Parasite Information Database (NEIHPID): Knowledge Base for Helminth Parasites

**DOI:** 10.1371/journal.pone.0157459

**Published:** 2016-06-10

**Authors:** Devendra Kumar Biswal, Manish Debnath, Graciously Kharumnuid, Welfrank Thongnibah, Veena Tandon

**Affiliations:** 1 Bioinformatics Centre, North-Eastern Hill University, Shillong, Meghalaya, India; 2 Department of Zoology, North-Eastern Hill University, Shillong, Meghalaya, India; 3 Biotech Park, Lucknow, Uttar Pradesh, India; Centre for Cellular and Molecular Biology, INDIA

## Abstract

Most metazoan parasites that invade vertebrate hosts belong to three phyla: Platyhelminthes, Nematoda and Acanthocephala. Many of the parasitic members of these phyla are collectively known as helminths and are causative agents of many debilitating, deforming and lethal diseases of humans and animals. The North-East India Helminth Parasite Information Database (NEIHPID) project aimed to document and characterise the spectrum of helminth parasites in the north-eastern region of India, providing host, geographical distribution, diagnostic characters and image data. The morphology-based taxonomic data are supplemented with information on DNA sequences of nuclear, ribosomal and mitochondrial gene marker regions that aid in parasite identification. In addition, the database contains raw next generation sequencing (NGS) data for 3 foodborne trematode parasites, with more to follow. The database will also provide study material for students interested in parasite biology. Users can search the database at various taxonomic levels (phylum, class, order, superfamily, family, genus, and species), or by host, habitat and geographical location. Specimen collection locations are noted as co-ordinates in a MySQL database and can be viewed on Google maps, using Google Maps JavaScript API v3. The NEIHPID database has been made freely available at http://nepiac.nehu.ac.in/index.php

## Introduction

Countries in the tropics or subtropics provide optimum conditions for the growth and propagation of helminth parasites and India, which is located in a tropical zone, possess a helminth fauna that is rich both in numbers and in variety. The majority of metazoan parasites known to invade vertebrate hosts belong to 3 phyla: Platyhelminthes (flatworms including Monogenea, Trematoda and Cestoda), Nematoda (roundworms) and Acanthocephala (spiny-headed worms). They are the causative agents of many debilitating and deadly ailments of humans and animals. Parasite zoonoses (diseases that are naturally transmitted between animals and humans) are an important public health problem worldwide, in both developed and developing countries. Phylum-level molecular phylogenetic analyses of Platyhelminthes have shown that the groups Cestoda, Monogenea and Trematoda form a clade [1,2,3]. Cestoda comprises more than 5000 species, many of which were described in ancient times. Molecular systematic investigations have revolutionised our understanding of cestode relationships and evolution [4,5,6,7,8]. The Digeneans comprise approximately 18,000 species, making them the largest group of metazoan zoonotic parasites. The classification, phylogeny and interrelationships of digenean taxa at both higher and lower levels remain unclear. Most of the families belonging to this group lack clear morphological features due to their complex life history involving a series of ontogenetic stages, hosts and ecological niches [9]. Of the three parasitic groups, Monogenea is the smallest, encompassing approximately 50% and 10% of the diversity of Cestoda and Digenea, respectively. Considerable effort will be required to catalogue the biodiversity of and determine the phylogenetic relationships among Indian and global Monogenea. The Indian Monogenoidea is well documented, primarily based on reviews by Chauhan (1953) and Yamaguti (1961). With 99 genera belonging to 21 families, and the need to thoroughly examine the hosts in the Indian subcontinent, cataloguing the biodiversity of the group is a large task [10,11,12].

The recent advances in molecular and medical helminthology provide tremendous scope for improvements in helminth therapeutics. There is a need to oversee the research strategies employed in helminthological science, as we are still at a relatively nascent stage in understanding helminth genomics and biodiversity. The biodiversity and bioinformatics databases pertaining to helminths are not keeping pace with the current enthusiasm shown by global researchers in helminthology. At present, in the absence of adequate support for helminthology training we risk losing the opportunity to recruit young scientists with varied skills to the study of parasite biology and associated diseases. Access to anthelminthic measures is now perceived as a human right, and consequently, previously disregarded helminthic illnesses must be addressed, and research into their treatment must be encouraged [13,14].

In the present database there is a focus on characterization of parasite biodiversity in mammalian livestock and other food animals in Northeast India by integration of comparative genomics and molecular systematics that encompass parasite primary specimens, host information, and spatial and temporal data. These are archived and housed in the proposed project with results of analyses, diagnostic capacity, images, etc., with synoptic summaries for parasite and host associations. These archives can form the basis for educational materials to the end users and researchers. Database development on health-based issues pertaining to those of parasite origin (eg. helminthic infections) and specific to North-East India would provide definitive information about the prevalence and preventive measures related to food-borne trematode and other helminthic infections. Parasite genomics research will help identify species-specific molecular markers; diagnostic probes as the basis for a predictive framework to understand patterns of parasite distribution and the potential for emergence and disease. The on-going three genome projects on *Fasciolopsis buski*, *Paragonimus westermani* and *Artyfechinostomum sufratyfex* have generated data sets that are complex in nature owing to their generation from modern sequencing techniques. Students with life sciences background now face the challenge of analysing high-throughput data. The present database captures the taxonomic hierarchy of helminth parasites, their habitat and biology along with diagnostic molecular markers feature information. Genome wide data pose relevant questions for representing large-scale data sets and bioinformatics ways to analyse these data. This will aid in innovative ways of bioinformatics applications and develop analytical approaches to extract biological information from large data sets. Biology students would derive the requisite impetus for exploring large-scale data and ways and means for analysing them through integrative bioinformatics applications. Our database of helminth parasites via informative tutorials provides just such a setting.

Globally, approximately 70 species of intestinal trematodes have been reported to infect humans [15]. The epidemiology of fish-borne zoonotic trematodiasis is complex because humans and reservoir hosts, such as dogs, cats, pigs, and fish-eating birds, harbour egg-shedding adult stages [16]. In addition to fish, crustaceans have been shown to be second intermediate hosts for a large number of digenetic trematodes, by harbouring their infective metacercarial larval stage [17, 18, 19]. The majority of food-borne trematodiases (FBTs) result from the consumption of raw or insufficiently cooked fish or crustaceans. It is only in recent years that this group of varying aetiology has begun to attract the interest of researchers. Thus, a general and concise compendium of the spectrum of helminth parasites in northeast India based on the presently available information has been compiled into a database (Helminth Parasite Spectrum in North-East India) at the Bioinformatics Centre, North Eastern Hill University, Shillong, India.

## Methods

The present dataset provides complete information on the species composition, host, distribution and taxonomic status of the helminth parasites in northeast India. Currently, information on 121 types of helminths (including platyhelminth, nematode and acanthocephalan parasites) that are known to occur in vertebrate hosts with food value (fish, amphibians, poultry, ruminants and pigs) in north-eastern India has been formatted and entered into the database. Information on additional taxa (e.g., parasites of rodent hosts) is being prepared and will also be included in the database. The database also includes annotated molecular sequences, on which motifs that can be used to distinguish many species (>100 isolates) of platyhelminth parasites have been noted. The database was developed using MS-Access and VB6.0; it is dynamic and will continue to be updated.

**Fig 1:**
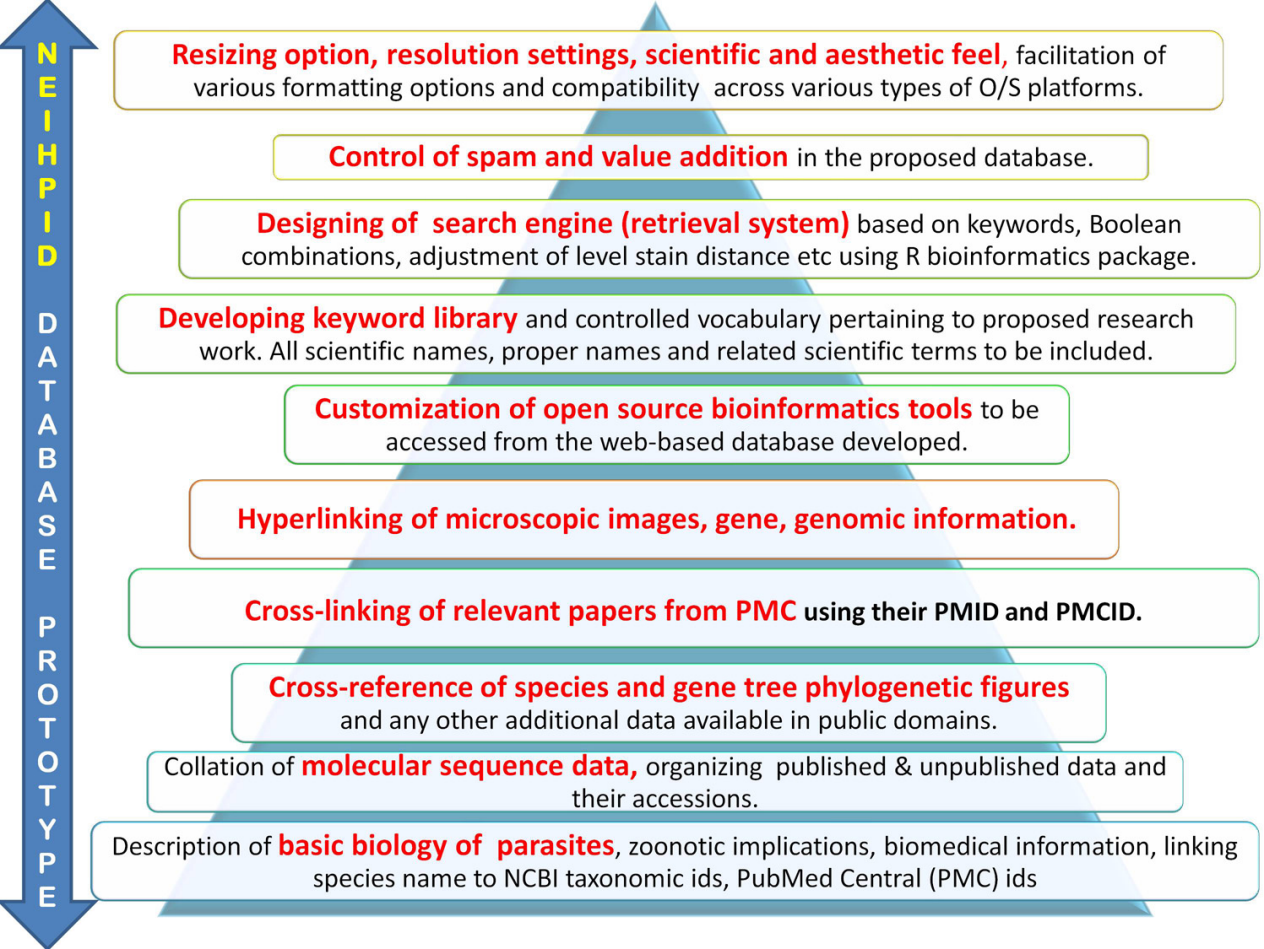
Overview of the NEIHPID database prototype.

In-silico studies of parasitic helminths (namely, trematodes: *Paragonimus* (lung flukes), *Fasciola* and other liver flukes, *Fasciolopsis* and other gastro-intestinal flukes; cestodes: *Taenia* and metacestodes (bladder worms); and nematodes: *Ascaris*, hookworms, filarial worms) have provided insights into how an organism’s characters or phenotype are determined by its genome sequence [20–22]. Experimental data generated by sequencing labs and made available in the public domain provide the basis for the systematic genomic analysis. With the advent of techniques for large-scale sequencing, many genome sequences of parasites are now available on the internet [23]. The relevant databases and web servers containing this information were searched for data that could be included in the present database. The analysis and interpretation of genomic data identified by searching the internet was compiled and relevant knowledge was derived with the aid of information and communication technologies (ICT) (Fig 1). Further, computational analysis was performed on genomic and extra-chromosomal regions, and identification of suitable markers therein, as a function of sequence divergence, provided data on the evolutionary trajectory of the organisms. In our present study, the complete mtDNA nucleotide sequence of *P*. *westermani*, which was collected from several sites in Changlang District, Arunachal Pradesh in India, was determined using total genomic DNA extracts from NGS data. A concatenated supermatrix of all the 12 protein-coding genes of mitochondrial DNA sequences of digenean trematode and cestodes, available in public domain (GenBank) was used for the phylogenetic analysis. Illumina reads from our unpublished *P*. *westermani* whole genome data were mapped to *P*. *westermani* reference sequence (gi|23957831| ref| NC_002354.2) and aligned using Bowtie aligner. Custom perl scripts were written to extract the mapped reads in fastq format. Assembly for the the Ion Torrent-mapped reads were performed using Newbler and Velvet software. Sanger reads were also added in the final assembly. Using Ion Torrent reads, Illumina reads, Sanger reads, hybrid high-quality de novo assembly the draft sequence was generated and finally the de novo-leftout regions were retrieved using reference assisted assembly and consensus calling. The complete sequence was generated with extensive manual curation work [24, 25].

In the first phase of gathering NGS and genomic data on parasites of medico-veterinary significance in northeast India, we have identified and undertaken the whole genome, transcriptome and mitochondrial sequencing of three trematodes: the lung fluke *Paragonimus westermani* and the intestinal flukes *Fasciolopsis buski* and *Artyfechinostomum surfratyfex*. The high-throughput raw data generated from these sequencing projects, which are currently being annotated, have been made available through the NEIHPID database web portal, subject to online registration in the database portal. To date, two mitochondrial genomes have been published [24,25].

### Database design and architecture

**Fig 2:**
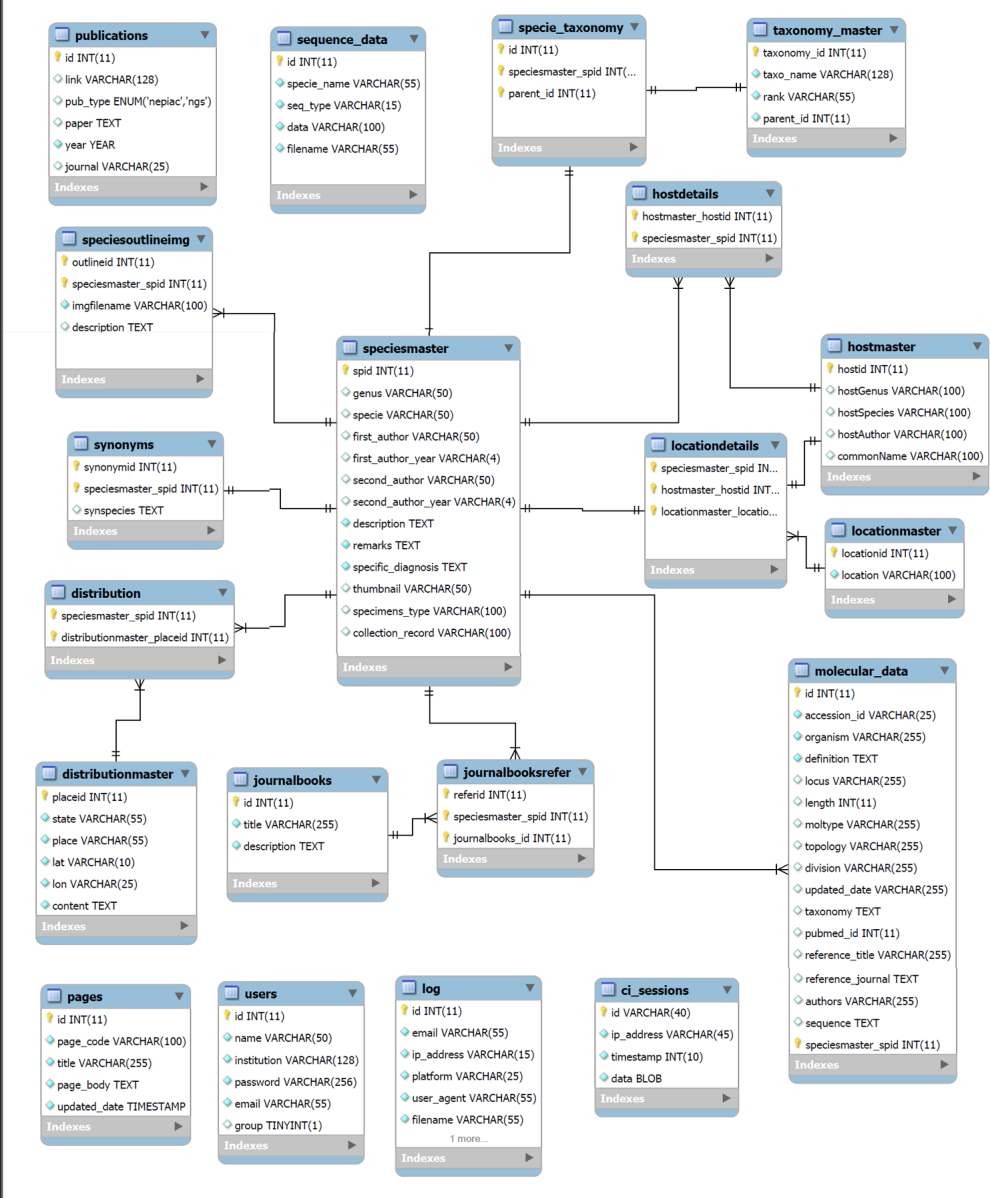
Extended Entity Relationship (EER) Diagram using MySQL Workbench 5.2.

The database design architecture is graphically presented in an Extended Entity Relationship (EER) Diagram using MySQL Workbench 5.2. At the back end, the NEIHPID database implements a cross-platform relational database management system (RDBMS), MySQL 5.5.24 for data storage and PHP 5.3.13 for writing and presenting dynamic web pages on the client browser. The application is hosted on an APACHE2.2 web server running the Red Hat Linux Enterprise Edition Operating System (RHEL6) (Fig 2).

The EER diagram contains various modules. Boxes show different tables (titles are listed at the top of the individual tables). Foreign keys between tables are shown. Some details of the model have been ignored to reduce diagram complexity.

The database facilitates storage of coordinate values along with geographical data on parasites collected from a particular site. The data can be plotted using Google Maps on an HTML page using Google Maps JavaScript API v3. The HTML page provides a list of parasites with their coordinate values, with hyperlinks to their morphological details, images, taxonomic hierarchy, synonyms, host, habitat, sequence data and important research findings. The database also includes NGS data and genomic data for parasites of medico-veterinary significance in northeast India. Data is available for download by registered users only.

**Fig 3:**
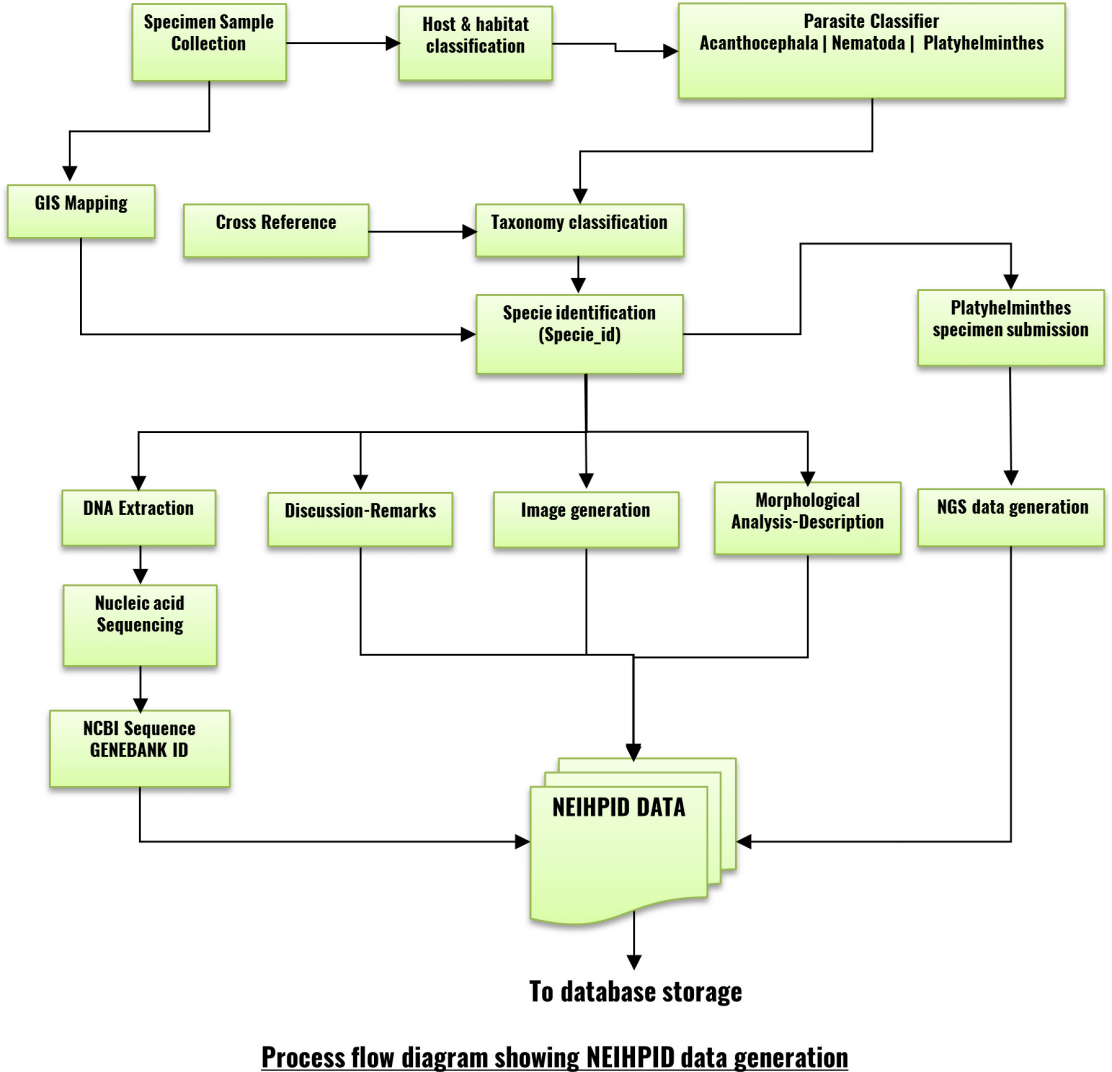
NEIHPID data integration and process flow diagram.

NEIHPID is a highly scalable database with the potential to expand to meet future demand. It currently contains six modules: (1) Geographical Information, which provides data on the collection sites of a parasite; (2) Host and Location, which details the host species and taxonomy, as well as the habitat of the parasite inside the host; (3) Image, which provides sketches or microscopic images of each parasite; (4) Taxonomy, which provides the taxonomic classification of each parasite; (5) Molecular, which provides information on associated parasite gene sequences, hyperlinked to GenBank at the National Centre for Biotechnology Information (NCBI) and (6) NGS, which provides NGS data and associated annotation for three selected platyhelminth parasites (Fig 3). Each module is designed to contain the maximum information about each parasite in order to deliver fast, accurate, efficient and reliable information on the web to end users.

### Database description

Each parasite entry contains information for each step, from collection to the laboratory, as follows:

#### Parasite

FoundAtPlaceName->InsideAHost->InsideAHostOrgan

>TakeImagesOfParasiteAtTheLab-

>ReferToJournalToAscertainFindings-

>ClassifyTaxonomyHierarchy->GenerateMolecularSequences

**Fig 4:**
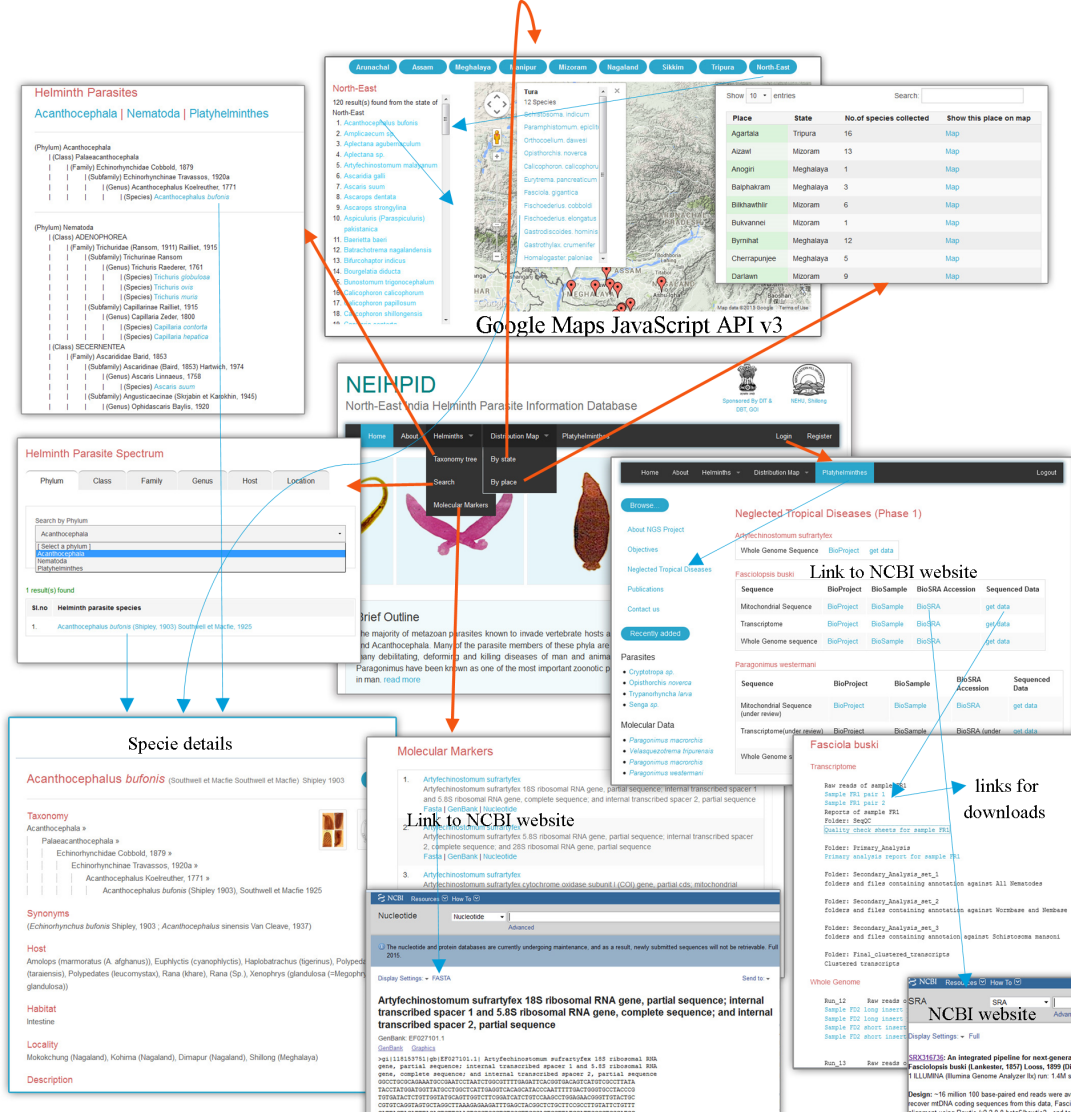
Screenshot and example of query results of the NEIHPID web interface.

Each parasite is associated with a collection locality and a host. Each parasite is studied by viewing it under a microscope. The researcher draws a sketch or takes a photograph of the parasite under a microscope. After studying it, the researcher may classify the parasite under a certain taxonomic hierarchy with reference to a book or journal article. The researcher may then generate molecular data, and annotate them with the aid of tools available at the NCBI or other public domain resources. We input this information into modular forms. End users can interactively browse the database using a web browser on an HTML page, which contains a Google Maps entry and useful data for querying a parasite entity. One can send queries to the server through client HTML pages embedded with JavaScript codes and a JavaScript library. The server side consists of an APACHE web server that handles user requests, while PHP modules effectively mediate between user queries and a MySQL database (Figs 3 and 4). Thus, queries can efficiently be processed by the NEIHPID database from any device, including smart phones, tablets, laptops and desktop computers that have a web browser installed. A detailed description of parasite taxonomy, at the species level, can be accessed hierarchically via the taxonomy tree. The taxonomy tree further provides links to a detailed account of the morphological studies, images and taxonomic hierarchy, synonyms, host, habitat and important research findings on the selected parasite (Fig 4).

#### Quality Check and Testing

Quality check of the NEIHID database was performed by using data generated from the study locations. The data was entered in all the PHP modules and validated at two stages: (i) web-page entry stage and (ii) MyAdmin database stage. The taxonomic data was published with actual voucher number catalogued by reputed in-house scientists working in the area. New taxa holotypes have been deposited in the national repository Zoological Survey of India, Kolkatta, India headquarters and paratypes of all these biological specimens are deposited in the departmental repository, Department of Zoology, North Eastern Hill University, Shillong, Meghalaya, India. Information from other workers are supplied in the remarks section of each taxon entry in the database.

## Results and Discussion

**Fig 5:**
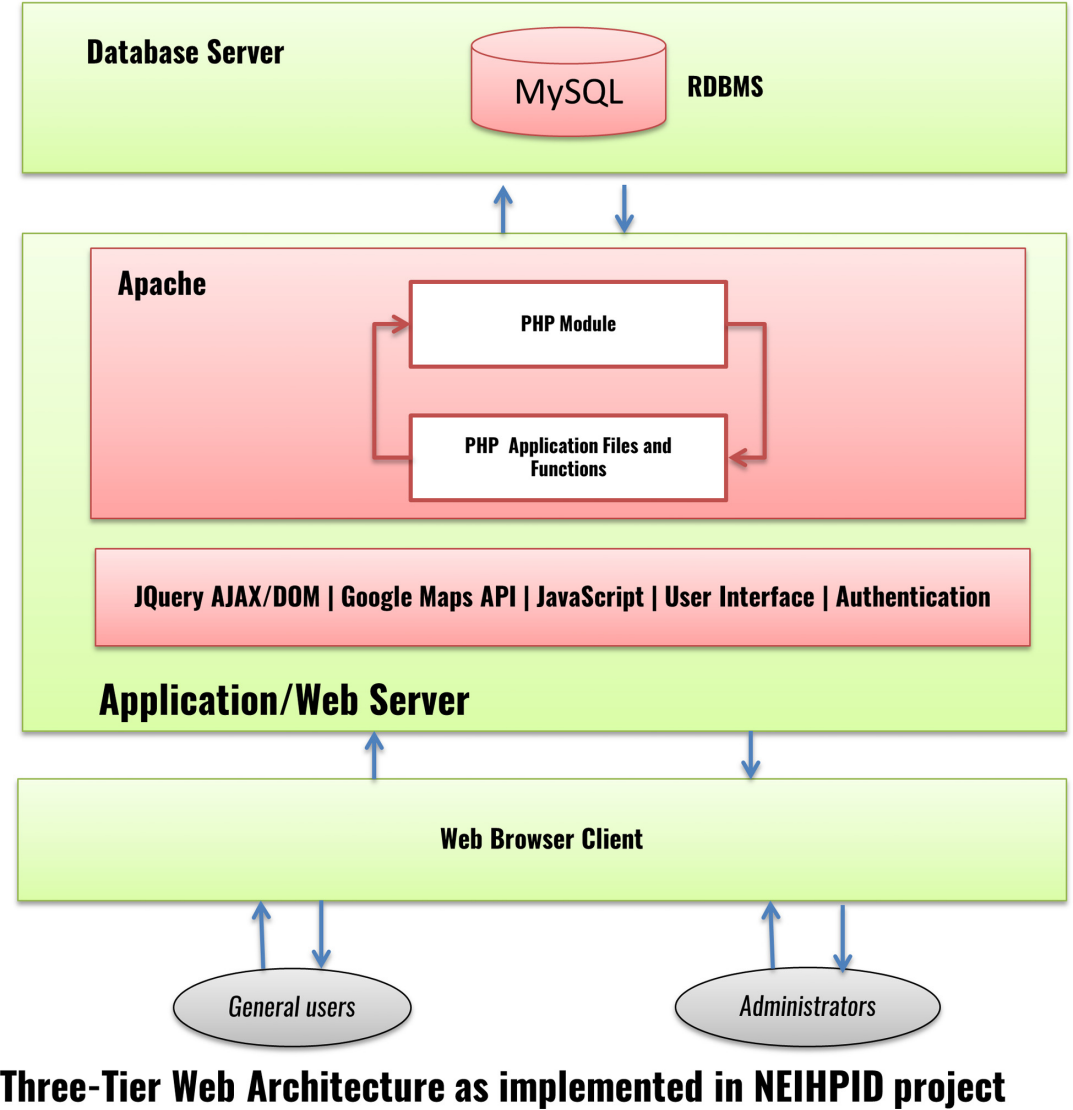
NEIHPID three-tier web architecture.

This database of parasites endemic to northeast India will provide a reference for studying the full spectrum of helminth parasites, especially those with zoonotic potential. The database contains information on classical taxonomy, morphology-based classification, disease information (if any), relevant references, and molecular sequences that serve as taxon (genus/species)-specific markers that are useful in diagnosis. The database will also provide study material for students interested in parasite biology. Users can search the database by species, host, or location, and collection sites are shown in Google maps using Google Maps JavaScript API v3. The NEIHPID was built on a RDBMS system based on three-tier architecture: client tier, middle tier and a database tier, suitable for large and scalable databases (Fig 5). Thus, the database can be scaled across a cluster of separate database servers, which can be accessed or reassembled efficiently. The database is also vertically scalable, i.e., additional capacity can be added to a single machine. A detailed tutorial on the operability and usage of the database is provided in the database link (http://nepiac.nehu.ac.in/helminth/page/tutorials.html) d the sae has been uploaded in google you tube.

The NEIHPID database was built in an RDBMS system based on three-tier architecture: client tier, middle tier and database tier, suitable for large and scalable databases.

### Search Options

#### A. Text-based searches

Text-based searches are implemented using JavaScript and jQuery. There are three optional group choices, and users can search by:

1. A predefined taxonomic level from the dropdown lists on the HTML page i.e. by phylum, class, family or genus.
2. Host (by genus name, subsequently filtered by species name).
3. State name (state name filtered by place name of collection locality).

#### B. Geographical Browsing

Geographical browsing is implemented using Google Maps JavaScript API v3 with jQuery. JQuery, which is a fast, small, and feature-rich JavaScript library, is widely used along with PHP as a general-purpose scripting language, especially meant for web development data retrieval from MySQL databases.

Our molecular sequence data are derived from primary databases (EMBL/GenBank/DDBJ) using the retrieval systems viz. SRS and Entrez and stored locally in our database.

#### Next generation sequencing data

**Fig 6:**
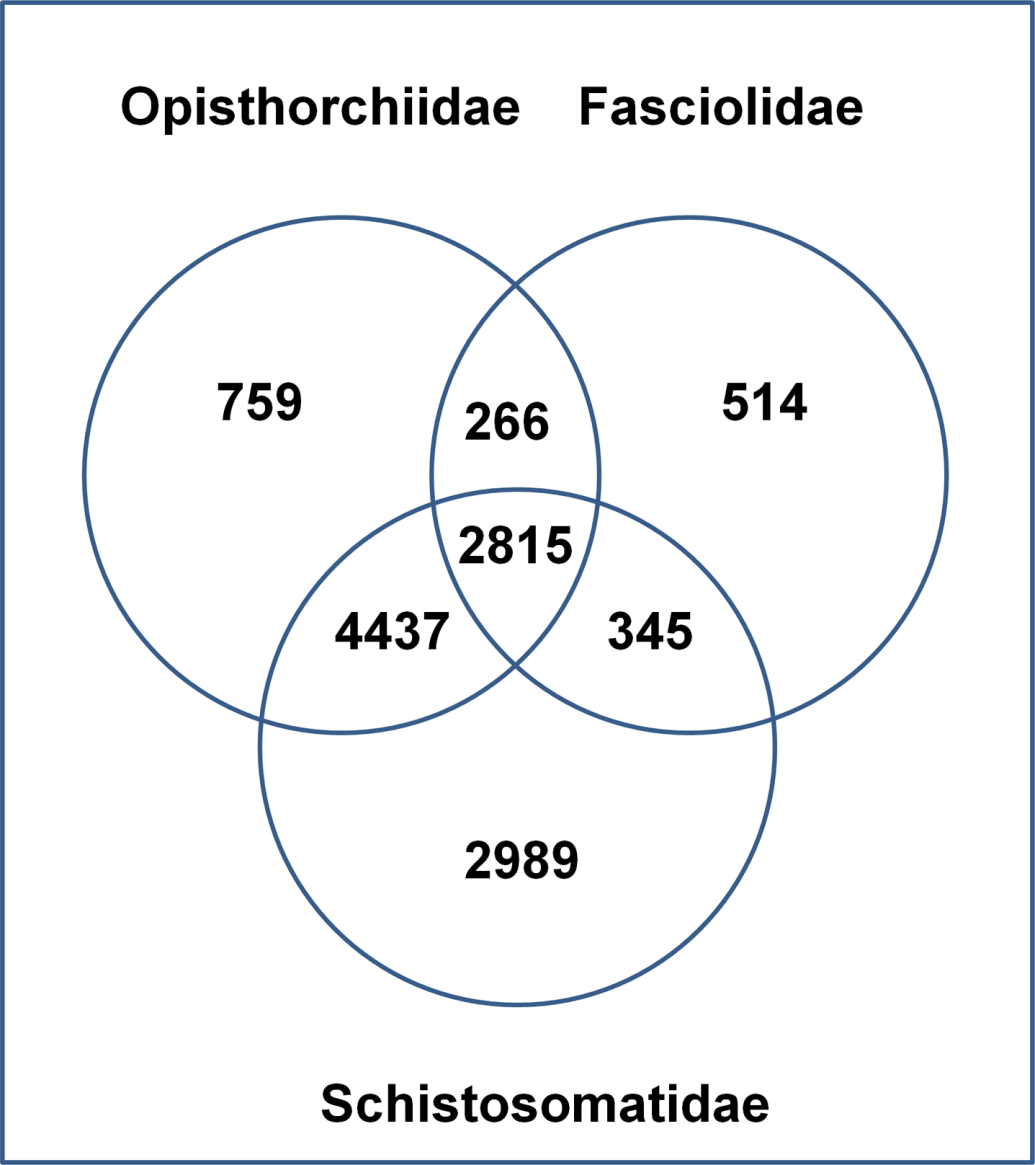
Venn diagram showing the number of homologs found in the *Fasciola buski* transcriptome compared to transcriptome/EST datasets of major trematode families of Opisthorchiidae (*Opisthorchis viverrini* and *Clonorchis sinensis*), Fasciolidae (*F*. *hepatica* and *Fasciola gigantica*) and Schistosomatidae (*Schistosoma mansoni* and *Schistosoma japonicum*).

**Fig 7:**
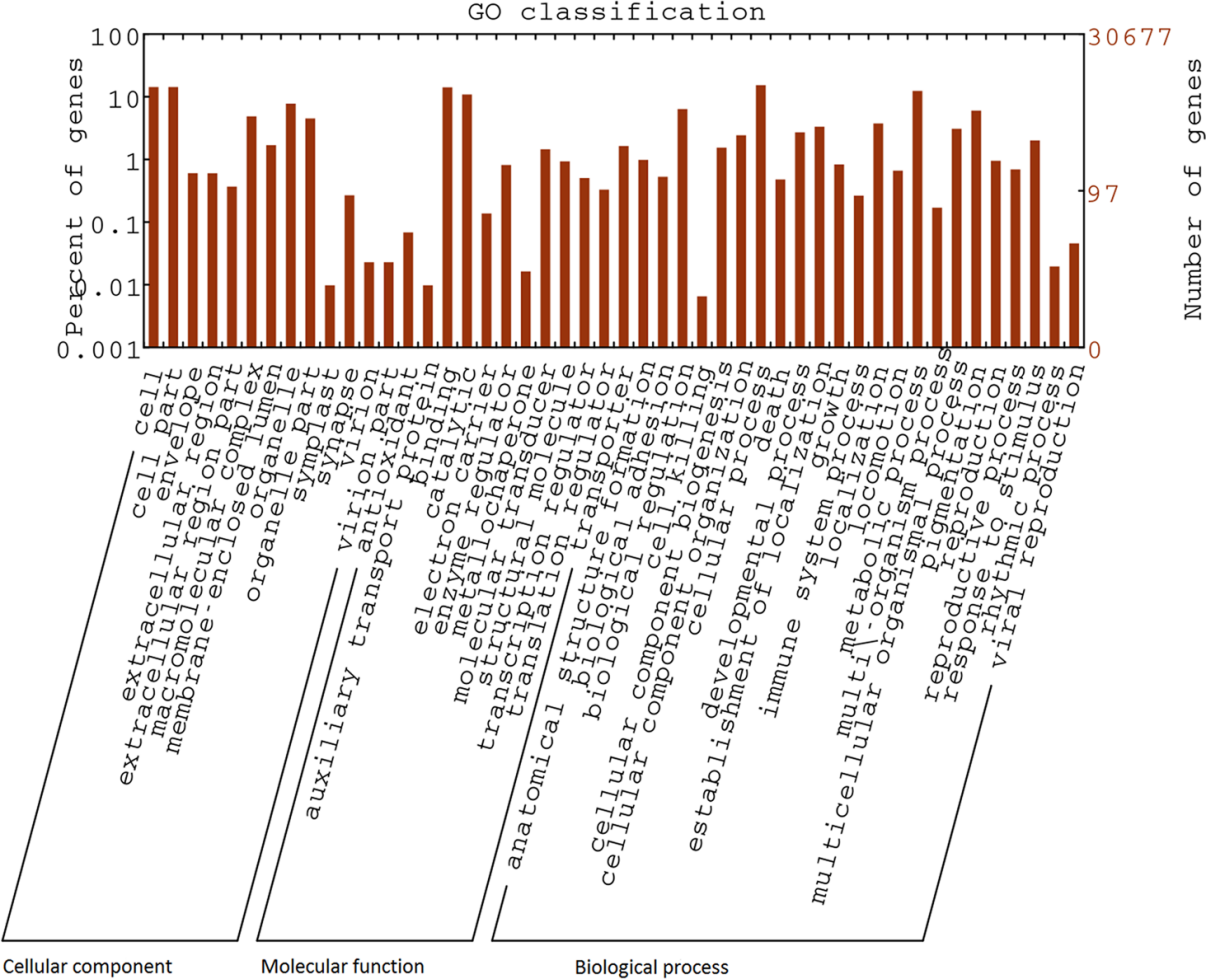
Gene ontology (GO) classification of the *Fasciola buski* transcriptome. GO terms assigned to unigenes were classified into three major functional classes: cellular components, biological processes and molecular functions.

The database contains the results of transcriptome analysis of the giant intestinal fluke, *Fasciolopsis buski*, obtained using NGS technology. Short-read sequences derived from polyA-containing RNA were assembled into 30677 unigenes, of which 12380 genes were annotated. Annotation of the assembled transcripts allowed analysis of various processes and pathways, such as RNAi pathways and energy metabolism. The expressed kinome of the organism was deciphered by identifying all protein kinases. We also performed genome sequencing and used the sequences to confirm the absence of some genes not observed in the transcriptome data, such as genes involved in fatty acid biosynthetic pathways. Transcriptome data also helped us to identify some of the transposable elements expressed. Though many long interspersed elements (LINEs) were identified, only two short interspersed elements (SINEs) were found. Transcriptome analysis revealed some of the biological characteristics of *F*. *buski* and provided an enormous resource for development of a suitable diagnostic system and therapeutic molecules (Figs 6 and 7). We established a high-throughput sequencing and bioinformatics pipeline for mitochondrial (mt) genomics for *F*. *buski* that emphasises the utility of short-read NGS platforms, such as Ion Torrent and Illumina, for sequencing and assembling the mt genome using innovative approaches for PCR primer design as well as assembly. Two different NGS platforms were employed for complete sequencing, assembly and annotation of the *F*. *buski* mt genome. The complete mt genome sequences of this intestinal fluke comprise only 14,118 bp; it is thus the shortest trematode mt genome sequenced to date [24]. These mtDNA NGS data will aid in investigating the taxonomy and systematics of the family Fasciolidae (Trematoda: Digenea) and will serve as a resource for comparative mitochondrial genomics and systematic studies of trematode parasites [24].

The complete mtDNA sequence (15,004 bp) of *P*. *westermani*, the Indian lung fluke and a major etiological agent of paragonimiasis, provides important genetic markers for ecological, population and biogeographical studies and molecular diagnosis of digeneans that cause trematodiases. The Ion Torrent NGS platform was harnessed to completely sequence the mt genome, and innovative approaches were applied to bioinformatically assemble and annotate it [25].

We achieved the following from the project with application of ICT in parasite genomics research:

- Creation of an integrated web-based NEIHPID database related to parasitology
- Characterisation of parasite biodiversity in mammalian livestock (cattle, sheep, goats, pigs, etc.) and other food animals (crustaceans, fish and poultry) in north-eastern India
- Identification of taxon-specific molecular markers for accurate diagnosis
- Comparative in-silico study of food-borne trematode and other helminthic infections
- NGS of selected trematodes and generation of transcriptome, whole genome and whole mt sequences

At present there are no vaccine or immunotherapy regimens in circulation for the treatment of any human parasitic infection and pharmaceutical approaches are alarmingly encountering parasitic drug resistance. The recent availability of sequences of several food-borne trematode and helminth parasites in the public domain via GenBank etc. has provided the opportunity to characterize novel antigens and metabolic enzymes essential for the parasite life cycle that might help in predicting novel therapeutic targets. Completed whole genome/ organelle genome sequences of some of the selected parasites (*P*. *westermani*, *F*. *buski* and *A*. *sufratyfex*) whose infections are zoonotic in nature have opened up avenues for carrying out post-genomic research. Comparisons can be performed within genomes and between genomes. Within genome comparisons will focus on analyzing variations in base composition, k-tuple frequency, gene density, variation in transposable elements, identification of any duplicated regions. Between-genome comparisons will employ closely related organisms (e.g., to identify conserved genes, gene organizations, and control elements) or more distant organisms (to identify genes that are confined to particular clades of a phylogenetic tree). Such data has helped trace synteny and gene order and evolutionary trajectories of organisms. Post-genome sequence analyses will attempt to confirm, support, and extend the genome annotation via hypothesis-based experimentation into the biological aspects of the parasite life cycle [20–25]. The project is novel because it marks the first time that the Department of Information Technology (DIT), Government of India (GOI) sponsored a life sciences project with an *in silico* approach. It aimed at characterising the parasite biodiversity unique to the region by capturing the various helminth parasite life forms with a focus on primary data analysis as well as the parasite data available in public domains. The outcome of the project is a collated compendium of enriched knowledge-base on parasite biology and its impact on human and social well-being, especially in northeast India.

### DNA Deposition

DNA sequences were deposited as follows:

National Centre for Biotechnology Information (NCBI) Bioproject database: PRJNA210017 and ID: 210017.

Bioproject: PRJNA248332, Biosample: SAMN02797822 and SRX550161

NCBI Sequence Read Archive (SRA): SRR924085.
